# Mirogabalin vs pregabalin for chemotherapy-induced peripheral neuropathy in pancreatic cancer patients

**DOI:** 10.1186/s12885-021-09069-9

**Published:** 2021-12-09

**Authors:** Mitsuru Sugimoto, Tadayuki Takagi, Rei Suzuki, Naoki Konno, Hiroyuki Asama, Yuki Sato, Hiroki Irie, Yoshinori Okubo, Jun Nakamura, Mika Takasumi, Minami Hashimoto, Tsunetaka Kato, Ryoichiro Kobashi, Takuto Hikichi, Hiromasa Ohira

**Affiliations:** 1 Department of Gastroenterology, School of Medicine, Fukushima Medical University, Fukushima, Japan; 2 Department of Endoscopy, Fukushima Medical University Hospital, Fukushima, Japan

**Keywords:** chemotherapy-induced peripheral neuropathy, mirogabalin, pregabalin, pancreatic cancer

## Abstract

**Background:**

The prognosis of pancreatic cancer (PC) has been improved by new chemotherapy regimens (combination of 5-fluorouracil, oxaliplatin, irinotecan, and leucovorin (FOLFIRINOX) or gemcitabine plus nab-paclitaxel (GnP)). Unfortunately, chemotherapy-induced peripheral neuropathy (CIPN) is a common adverse event of these two regimens. The efficacy of pregabalin for CIPN has been reported in previous studies. However, the efficacy of mirogabalin for CIPN remains unknown. Thus, in this study, we aimed to clarify which drug (mirogabalin or pregabalin) was more valuable for improving CIPN.

**Methods:**

A total of 163 PC patients who underwent FOLFIRINOX or GnP between May 2014 and January 2021 were enrolled. Among them, 34 patients were diagnosed with CIPN. Thirteen patients were treated with mirogabalin (mirogabalin group), and twenty-one patients were treated with pregabalin (pregabalin group). Treatment efficacy was compared between the two groups.

**Results:**

In both the mirogabalin group and the pregabalin group, the grade of patients with CIPN at 2, 4, and 6 weeks after the initiation of treatment showed significant improvement compared to the pretreatment grade. Notably, the rate of CIPN improvement was higher in the mirogabalin group than in the pregabalin group (2 weeks: 84.6% (11/13) vs 33.3% (7/21), *P* value = 0.005; 4 weeks, 6 weeks: 92.3% (12/13) vs 33.3% (7/21), *P* value = 0.001).

**Conclusions:**

Although both mirogabalin and pregabalin were effective at improving CIPN, mirogabalin might be a suitable first choice for CIPN in PC patients.

**Trial registration:**

Not applicable

## Background

Pancreatic cancer (PC) is a lethal disease that has become a major cause of cancer-related death worldwide [1–3]. The poor prognosis of most PC patients is due to the advanced stage of the disease at diagnosis, making resection difficult [4–6]. Thus, chemotherapy has become the general treatment strategy for PC patients. Recently, new chemotherapy regimens have been developed, such as the combination of 5-fluorouracil, oxaliplatin, irinotecan, and leucovorin (FOLFIRINOX) or gemcitabine plus nab-paclitaxel (GnP). Although the prognosis of PC patients is very poor, it has been dramatically improved by FOLFIRINOX or GnP [7–35]. On the other hand, many adverse events are also reported for these regimens. Chemotherapy-induced peripheral neuropathy (CIPN) is a common side effect of both FOLFIRINOX and GnP. In past reports, the frequency of grade 3-4 CIPN was 0 - 25% for FOLFIRINOX [7–9, 11, 12, 14, 15, 17, 18, 21–23, 36] and 1.8 – 30.4% for GnP [27–31, 35, 37]. Oxaliplatin and paclitaxel represent a class of neurotoxic drugs [38–40]. When CIPN becomes severe, it could influence the decision to continue chemotherapy, affecting patient prognosis. Therefore, adequate management of CIPN is necessary.

Regarding drug treatments for CIPN, the efficacy of duroxetine was demonstrated in a past large double-blind randomized controlled trial [41]. In addition, pregabalin was found to be more valuable for treating CIPN than duroxetine in some reports [42, 43]. On the other hand, the efficacy of mirogabalin for diabetic peripheral neuropathy has also been reported [44]. Recently, mirogabalin treatment for CIPN was covered by medical insurance in Japan. Unfortunately, the efficacy of mirogabalin for CIPN is unknown. Therefore, in this study, we compared mirogabalin and pregabalin for the treatment of CIPN.

## Methods

### Study design and ethics

This was a retrospective study comparing the efficacy of mirogabalin and pregabalin for the treatment of CIPN. This study was approved by the Institutional Review Board of Fukushima Medical University (approval number: 29254). The analysis used anonymous clinical data obtained after all the participants agreed to treatment by written consent, so patients were not required to give informed consent for the study. Informed consent was obtained from all participants or, if participants were under 18, from a parent and/or legal guardian. The details of the study can be found on the homepage of Fukushima Medical University. All methods were carried out in accordance with relevant guidelines and regulations.

### Patients

**Fig. 1:**
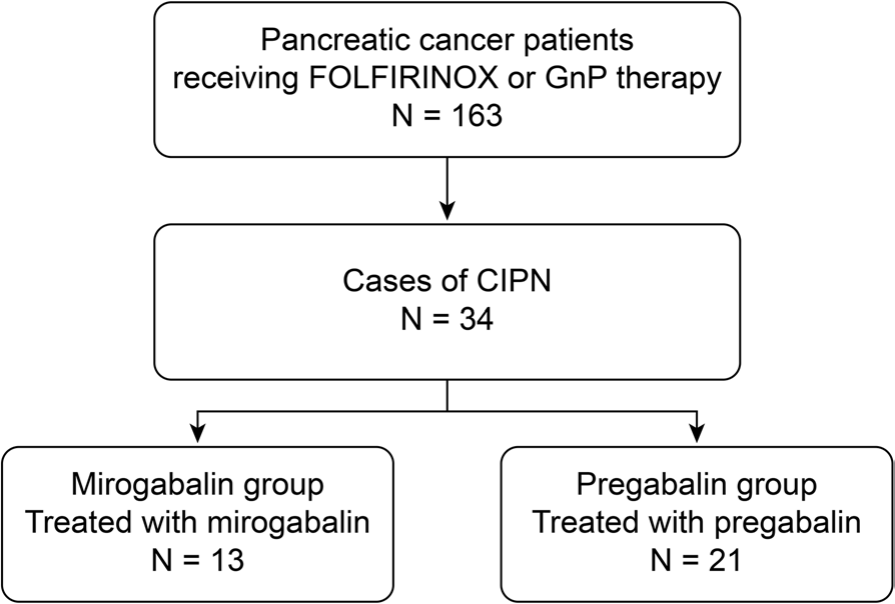
Flowchart of patient selection

A total of 163 PC patients who underwent FOFIRINOX or GnP therapy at Fukushima Medical University between May 2014 and January 2021 were enrolled. Among them, 34 patients were diagnosed with CIPN based on its clinical course. When a PC patient who was administered a neurotoxic drug reported new pain or numbness on the extremities, the patient was diagnosed with CIPN [45]. Thirteen patients were treated with mirogabalin (mirogabalin group), and twenty-one patients were treated with pregabalin (pregabalin group) (Fig. 1). PC was diagnosed by endoscopic ultrasonography-guided fine needle aspiration, abdominal ultrasonography-guided biopsy, bile cytology, or biliary biopsy.

### Dose of mirogabalin, pregabalin

The dosages of mirogabalin and pregabalin were determined by each doctor, and the effect of mirogabalin or pregabalin was evaluated every one or two weeks. If CIPN did not improve, the dose of mirogabalin or pregabalin was increased. On the other hand, when a side effect of mirogabalin or pregabalin was observed, the dose was decreased. When a side effect became severe or CIPN was sufficiently improved, mirogabalin or pregabalin was stopped. The actual dosage of mirogabalin was as follows (10 mg/day for 4-6 weeks: six patients, 10 mg/day for a week→20 mg/day for a week→30 mg/day for four weeks: a patient, 10 mg/day for a week→ 15 mg/day for a week→20 mg/day for four weeks: a patient, 5 mg/day for a week→15 mg/day for two weeks→10 mg/day for a week→20 mg/day for two weeks: a patient, 10 mg/day for two weeks→20 mg/day for four weeks: a patient, 10 mg/day for four weeks→20 mg/day for two weeks: a patient, 10 mg/day for a week→20 mg/day for five weeks: a patient, 5 mg/day for two weeks→10 mg/day for four weeks: a patient). The actual dosage of pregabalin was as follows (150 mg/day for 2-6 weeks: 18 patients, 75 mg/day for 6 weeks: a patient, 150 mg/day for three weeks→75 mg/day for three weeks: a patient, 150 mg/day for four weeks→75 mg for two weeks: a patient).

### Examination items

Patient characteristics and background (age, sex, tumor stage based on the Union for International Cancer Control (UICC) classification 8^th^ edition [46], neurotoxic regimen, concomitant drugs for CIPN, pretreatment CIPN grade) were compared between the mirogabalin group and the pregabalin group. CIPN grade was compared between pretreatment and at 2 weeks after treatment, 4 weeks after treatment, or 6 weeks after treatment. The grade of CIPN was classified by Common Terminology Criteria for Adverse Events (CTCAE) version 5.0. The number of patients with improvement in CIPN at 2, 4, or 6 weeks after treatment was compared between the mirogabalin group and the pregabalin group. If a patient stopped taking medicine for several reasons (CIPN was sufficiently improved, drugs were ineffective, adverse events), the evaluation of improvement in CIPN was continued until 6 weeks after drug initiation.

### Statistical analysis

Continuous variables following a normal distribution (age) were analyzed with Welch’s t-test. Ordinal variables and continuous variables that did not follow a normal distribution were analyzed with the Mann-Whitney U test. Nominal variables were analyzed with Fisher’s exact test. The treatment effect of each group was compared between pretreatment and posttreatment with the Wilcoxon signed-rank test. *P* < 0.05 was defined as statistically significant. All statistical analyses were performed using EzR (Saitama Medical Centre, Jichi Medical University, Saitama, Japan).

## Results

### Patient characteristics and clinical background

Age, sex, UICC stage, neurotoxic regimen, and concomitant drugs for CIPN were not different between the two groups (Table 1). In both groups, the majority of patients underwent GnP. The pretreatment CIPN grade was significantly higher in the mirogabalin group than in the pregabalin group (3 (2-3) vs 2 (2-3), *P* < 0.01).

**Table 1:** Comparison of patient characteristics and clinical background

	Mirogabalin group (***N*** = 13)	Pregabalin group (***N*** = 21)	***P*** value
Age, years	61.3 ± 13.6	65.4 ± 7.2	0.33
Sex, male/female	8/5	9/12	0.48
UICC stage, median (range)	4 (3-4)	4 (2-4)	0.74
II, n (%)	0 (0)	1 (4.8)	
III, n (%)	5 (38.5)	8 (38.1)	
IV, n (%)	8 (61.5)	12(57.1)	
Neurotoxic regimen, n (%)			0.68
FOLFIRINOX	2 (15.4)	5 (23.8)	
GnP	11 (84.6)	16 (76.2)	
Concomitant drugs for CIPN, n (%)	3 (23.1)	5 (23.8)	1.0
Duroxetine	1 (7.7)	1 (4.8)	
Vitamin B12	2 (15.4)	1 (4.8)	
Duroxetine, goshajinkigan		1 (4.8)	
Goshajinkigan		2 (9.5)	
Pretreatment CIPN grade, median (range)	3 (2-3)	2 (2-3)	< 0.01
2, n (%)	1 (7.7)	13 (61.9)	
3, n (%)	12 (92.3)	8 (38.1)	

Values are shown as the mean ± standard deviation, median (range) or n (%)

*UICC* Union for International Cancer Control classification; *FOLFIRINOX* combination of 5-fluorouracil, oxaliplatin, irinotecan, and leucovorin; *GnP* gemcitabine plus nab-paclitaxel; *CIPN* chemotherapy-induced peripheral neuropathy

### Treatment effect for CIPN

**Fig. 2:**
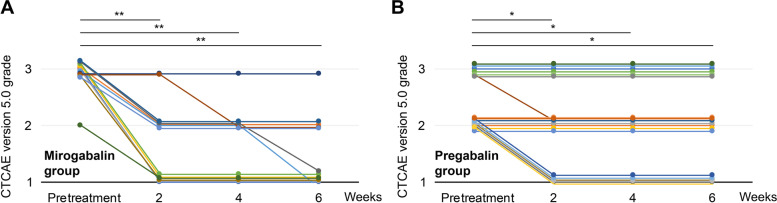
Grade of CIPN before and after treatment. **a**, **b**, The grade of CIPN at 2, 4, and 6 weeks after treatment initiation showed significant improvement compared to that before treatment initiation in both groups. *CIPN*, chemotherapy-induced peripheral neuropathy; *CTCAE*, Common Terminology Criteria for Adverse Events. * *p*<0.05, ** *p*<0.01

CIPN showed improvement in both the mirogabalin group and pregabalin group (Figure 2). In each group, the grade of CIPN at 2 weeks, 4 weeks, and 6 weeks after drug initiation showed significant improvement over the pretreatment grade.

**Fig. 3:**
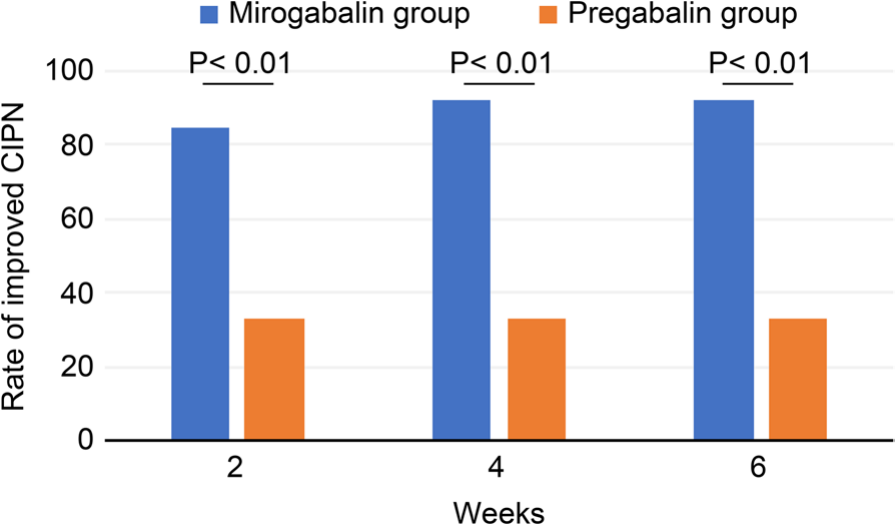
Rate of improvement in CIPN. The rates of improvement in CIPN at 2, 4, and 6 weeks after treatment initiation were significantly higher in the mirogabalin group than in the pregabalin group. *CIPN*, chemotherapy-induced peripheral neuropathy

The rate of improvement in CIPN at 2, 4 or 6 weeks after drug initiation was significantly higher in the mirogabalin group than in the pregabalin group (2 weeks: 84.6% (11/13) vs 33.3% (7/21), *P* value = 0.005; 4 weeks, 6 weeks: 92.3% (12/13) vs 33.3% (7/21), *P* value = 0.001) (Fig. 3).

### Drug discontinuation and adverse events

Drug discontinuation are shown in Table 2. Mirogabalin was stopped in two (15.4%) patients 4 weeks after it was initiated; one patient reported dizziness, and CIPN was found to improve very well in the other patient. Pregabalin was stopped in eleven (52.4%) patients. Seven (33.3%) patients stopped taking pregabalin 2 weeks after pregabalin was initiated. Sufficient improvement in CIPN was not observed in two patients, and side effects were observed in the other five patients. Four (19.0%) patients stopped taking pregabalin four weeks after pregabalin was initiated. Sufficient improvement in CIPN was not observed in three patients, and CIPN was found to improve very well in the other patient. The change in CTCAE grade was not observed after drug discontinuation.

**Table 2:** The reasons for drug discontinuation

Reason for drug discontinuation, n (%)	Mirogabalin group (*N* = 13)	Pregabalin group (*N* = 21)
2 weeks after initiation	0 (0)	7 (33.3)
Ineffective	0 (0)	2 (9.5)
Adverse events	0 (0)	5 (23.8)
4 weeks after initiation	2 (15.4)	4 (19.0)
CIPN sufficiently improved	1 (7.7)	1 (4.8)
Adverse events	1 (7.7)	0 (0)
Ineffective	0 (0)	3 (14.3)

Values are shown as n (%)

*CIPN*, chemotherapy-induced peripheral neuropathy

Adverse events were not significantly different between the mirogabalin group and the pregabalin group (Table 3).

**Table 3:** The comparison of adverse events

	Mirogabalin group (*N* = 13)	Pregabalin group (*N* = 21)	***P*** value
Adverse events, n (%)	2 (15.4)	7 (33.3)	0.43
Dizziness	1 (7.7)	2 (9.5)	
Edema	1 (7.7)	2 (9.5)	
Sleepiness	0	3 (14.3)	

Values are shown as n (%)

## Discussion

In this study, the majority of CIPN occurred by GnP. The therapeutic effect for CIPN was compared between mirogabalin and pregabalin. Both drugs were effective at improving CIPN in PC patients. Although the effect of mirogabalin on CIPN was unknown, the rate of improved CIPN was significantly higher in the mirogabalin group than in the pregabalin group.

In a recent meta-analysis that compared treatment outcomes between FOLFIRINOX and GnP, CIPN occurred much more frequently in patients who were treated with GnP than in patients who were treated with FOLFIRINOX [47]. Therefore, it was reasonable that the regimen responsible for most CIPN was GnP in this study.

Several drugs have been reported for treating CIPN. In these reports, calcium and magnesium, goshajinkigan, duloxetine, vitamin B12, pregabalin, and gabapentin were used [41–43, 48–62]. However, calcium/magnesium, goshajinkigan, and gabapentin were found to be ineffective at treating CIPN in the largest double-blind randomized controlled trials (RCTs) for each drug. Vitamin B12 was used as a control group in the study to investigate the efficacy of goshajinkigan and duroxetine. In the largest RCT, duroxetine was found to be effective at treating CIPN. In addition, the efficacy of pregabalin for CIPN was reported to be better than that of duroxetine in two reports. In 2018, Avan et al. [42] performed a double-blind RCT that targeted 82 breast cancer patients with taxane-induced peripheral neuropathy (pregabalin group: n = 40, duroxetine group: n = 42). In their study, pregabalin provided the greatest improvement in insomnia and pain scores [42]. In 2019, Salehifar et al. [43] reported that pregabalin was more valuable for improving the sensory and pain scores of CIPN than duroxetine. In both reports, CIPN was improved after 6 weeks of pregabalin treatment. In this study, CIPN was significantly improved after pregabalin treatment. Although mirogabalin was reported to be useful for diabetic neuropathy [44], it was also found to be useful for CIPN in this study.

Although mirogabalin and pregabalin were both valuable for improving CIPN, the treatment effect was different between the two groups. Although no significant difference in adverse events was observed, adverse events were more common in the pregabalin group than in the mirogabalin group. Pregabalin and mirogabalin combine with the α2δ subunit of Ca channels in the back horn of the spinal cord and impede the inflow of calcium, which is required for neurotransmitter release [44, 63–66]. However, the two drugs show different connectivity to the subtypes of the α2δ subunit of the Ca channel. Among these subtypes, the α2δ-1 subunit is related to analgesic effects [67], and the α2δ-2 subunit is related to central nervous system disorders [68]. In a past report written by Domon et al. [69], the dissociation half-life between mirogabalin and the α2δ-1 subunit was 11.1 (8.3-16.4) hours, and the dissociation half-life between mirogabalin and the α2δ-2 subunit was 2.4 (2.1-2.8) hours. On the other hand, the dissociation half-life between pregabalin and α2δ-1 and α2δ-2 subunits was 1.4 hours (α2δ-1: 1.4 (1.3-1.4) hours, α2δ-2: 1.4 (0.9-2.7) hours) [69]. Because the dissociation half-life between mirogabalin and the α2δ-1 subunit was longer than that between mirogabalin and the α2δ-2 subunit, the analgesic effect is expected to be durable, and adverse events caused by central nervous system disorder are expected to be reduced by mirogabalin. This difference in the connection to the α2δ subunit leads to differences in not only the treatment effects but also the adverse events elicited by mirogabalin and pregabalin.

There were some limitations to this study that should be mentioned. First, this was a retrospective study with a small sample size conducted at a single institution. However, this study is the first to compare the efficacy of mirogabalin and pregabalin for the treatment of CIPN. We hope that multicenter RCTs will be conducted in the future to confirm the results reported in this study. Second, the doses of mirogabalin and pregabalin were not uniform. The results showed that both drugs were effective at treating CIPN, even though a low dose was used for both drugs.

## Conclusions

Although both mirogabalin and pregabalin were effective at improving CIPN, a higher rate of improved CIPN was observed in patients who were treated with mirogabalin. Mirogabalin might be a suitable first choice for CIPN in PC patients.

## Data Availability

The datasets generated and/or analyzed during the current study are available from the corresponding author upon reasonable request.

## Abbreviations

PC: Pancreatic cancer
FOLFIRINOX: The combination of 5-fluorouracil, oxaliplatin, irinotecan, and leucovorin
GnP: Gemcitabine plus nab-paclitaxel
CIPN: Chemotherapy-induced peripheral neuropathy
